# Liposomal Honokiol induces ROS-mediated apoptosis via regulation of ERK/p38-MAPK signaling and autophagic inhibition in human medulloblastoma

**DOI:** 10.1038/s41392-021-00869-w

**Published:** 2022-02-21

**Authors:** Shenglan Li, Jinyi Chen, Yaqiong Fan, Ce Wang, Can Wang, Xiaohong Zheng, Feng Chen, Wenbin Li

**Affiliations:** Department of Neuro-oncology, Cancer Center, Beijing Tiantan Hospital, Capital Medical University, Beijing, China

**Keywords:** CNS cancer, Cell death in the nervous system, CNS cancer

**Dear Editor**,

Nowadays, medulloblastoma accounts for approximately 25% of all pediatric brain tumors.^1^ Even with the successful treatment of primary tumors, there are still serious side effects. Honokiol (HNK) is defined as small bisphenol lignin extracted from the bark and seed cones of several species of *Magnolia*. It has shown that HNK can inhibit the proliferation and promote the apoptosis of tumor cells, thereby exerting a potent antitumor effect.^2^ However, the poor water solubility of HNK results in its low bioavailability, thus limiting its wide use in clinical cancer treatments. Liposomes can overcome this limitation, and liposomal HNK (Lip-HNK) has promising clinical applications in this aspect. Currently, there is no study focusing on the treatment of medulloblastoma with HNK. Therefore, we explored the effect of Lip-HNK and its mechanism on medulloblastoma cells. Our findings may provide evidence of the potential of Lip-HNK as a new therapeutic agent for medulloblastoma.

In our study, it was found that increased Lip-HNK concentration could inhibit the proliferation of DAOY and D283 cells, without exerting effects on the growth of non-tumor cells (Fig. 1a). As the dose of Lip-HNK was increased, more G1 subsets were produced from DAOY and D283 cells (Supplementary Fig. 1a–d). Moreover, the level of P53 and P21 proteins (inhibiting cell cycle progression) was increased. Lip-HNK also downregulated the expression of CDK4 and cyclin D1 (Fig. 1b, c). Hoechst 33342 and PI staining were applied for the detection of cell apoptosis, with cells emitting a fluorescent signal considered dead cells, and Hoechst-only-stained cells counted as living cells (Supplementary Fig. 2a/b). The increase of Lip-HNK doses during treatment of DAOY and D283 cells caused more apoptotic cells (Supplementary Fig. 2c/d). The molecular mechanism of Lip-HNK-inducing the apoptosis was caspase-dependent (Supplementary Fig. 2e/f). Moreover, Lip-HNK caused apoptosis and death, which, in turn, led to the failure of mitochondrial membrane function (Supplementary Fig. 2g).

**Fig. 1:**
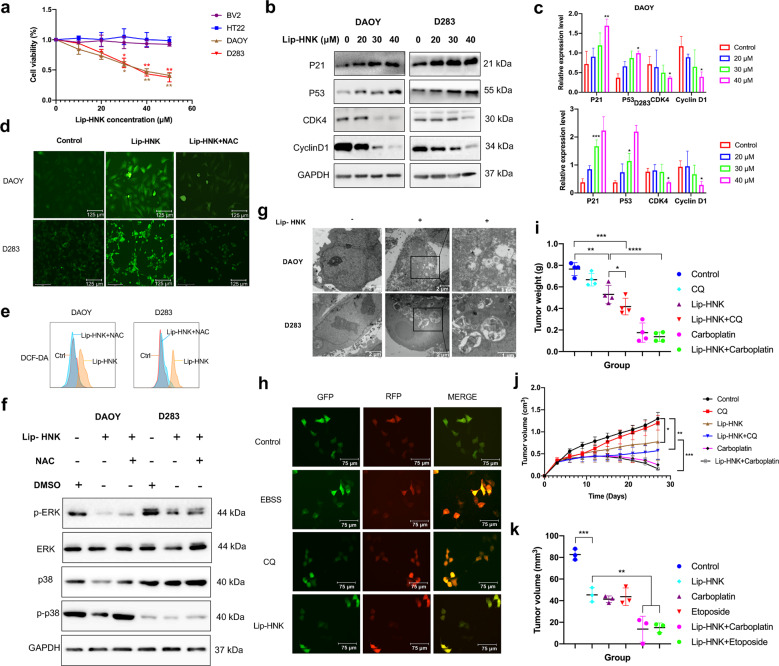
**a** D283, DAOY, BV2, and HT22 cells were treated with different concentrations of Lip-HNK. **b**, **c** Western blotting was used to detect protein levels of P21, P53, CDK4, and Cyclin D1 after treatment of Lip-HNK for 48 h. **d** ROS production was measured by DCFH-DA staining using a fluorescence microscope. **e** The influence of NAC on HNK-induced ROS production was observed by flow cytometry measurement. **f** Protein levels of p-ERK, ERK, p-p38, and p38 in medulloblastoma cells were detected after the combined treatment of 40 μM Lip-HNK and 5 mM NAC. **g** The subcellular morphology of DAOY and D283 cells treated with Lip-HNK for 48 h was examined using a transmission electron microscope. **h** DAOY cells with stable mRFP-GFP-LC3B expression were treated with the autophagy inhibitor (CQ), exposed to Lip-HNK or EBSS, fixed, and imaged with a fluorescence microscope. **i** The weight of tumor was represented by a scatter plot. **j** Tumor growth curves represented the average values of 4 mice in each group. **k** The tumor volume of individual mice was analyzed using soft of Dicom and calculated using the formula VM = length(max) × width × thickness. Data were presented as mean ± SD. **p < 0.01, ***p < 0.001 versus control

Mitochondria-generated intracellular ROS can affect the apoptosis of various cells.^3^ In our study, Lip-HNK induced ROS production, which, as hypothesized, was blocked by the ROS scavenger NAC (Fig. 1d–e). According to CCK-8 analysis, after NAC pretreatment, Lip-HNK-induced cell death was remarkably reduced (Supplementary Fig. 3a/b). Moreover, it was observed that Lip-HNK and NAC treatment could notably reduce annexin V-positive cells (Supplementary Fig. 3c). From all above, the production of ROS was related to the Lip-HNK-induced apoptosis of human medulloblastoma cells.

The influence of Lip-HNK on the ERK/p38-MAPK signaling pathway was explored for better understanding its anticancer mechanism (Supplementary Fig. 4a). NAC co-treatment could partially reverse ERK and p38 proteins (Fig. 1f). Moreover, the caspase-3 sectioned and the Bax protein level increased by Lip-HNK were also impacted and partially reduced. In contrast, when NAC was used as a co-treatment, caspase-3 and Bcl-2 protein levels were increased (Supplementary Fig. 4b). The above statistics indicated that excessive ROS not only affected Lip-HNK-induced apoptosis, but also was linked to the inhibition of the ERK/p38-MAPK signaling pathway.

Multiple studies have evidenced the interaction between autophagy and apoptosis in various cancer cells. In our study, it was noticeably observed that both APs and ALs were accumulated in DAOY and D283 cells under a transmission electron microscope after Lip-HNK treatment (Fig. 1g). Generally, the shift from LC3BI to LC3BII is a hallmark of autophagy.^4^ We found that LC3BII protein in the Lip-HNK-treated group was noticeably elevated (Supplementary Fig. 5a/b). Beclin-1 (BECN), Atg7 proteins, and LC3BII were dramatically upregulated in the Lip-HNK-treated cells (Supplementary Fig. 5c–f). These results suggested that Lip-HNK treatment induced early autophagy and its volume aggregation in the medulloblastoma cells.

The rise of autophagosomes may be due to the increase of autophagosome synthesis in the early stage or the inhibition of lysosome degradation in the late stage. In our study, Western blotting results revealed that Lip-HNK treatment remarkably increased p62 expression, which was dose-dependent (Supplementary Fig. 6a/b). These results confirmed that Lip-HNK treatment could induce the buildup of autophagosome via inhibiting the autophagosome degradation in the medulloblastoma cells. Chloroquine (CQ), as a specific late autophagy inhibitor, can inhibit the autophagy flux by prohibiting the maturation of autophagosome, thereby suppressing its progression to the degradable autophagosome.^5^ The tandem fluorescence-tagged LC3B (monomeric red fluorescence protein mRFP-GFP-LC3B protein; tfLC3) was used to confirm whether Lip-HNK treatment could inhibit the late-stage autophagy and then cause p62 accumulation. This probe capitalized on the pH difference between the autolysosome (acidic) and autophagosome (neutral), which denoted the autophagic flux from the autophagosomes (GFP^+^ RFP^+^; yellow dots) to the autolysosomes (GFP^−^ RFP^+^; red dots). It was found that the number of red dots in EBSS (positive control) was increased (Fig. 1h), which indicated an increase of autophagy flux. Lip-HNK treatment caused the increased formation of yellow puncta in CQ (10 μM) and DAOY cells.

To assess the effect of CQ combined with Lip-HNK on medulloblastomas in vivo, we induced the xenograft tumor models via subcutaneously inoculating DAOY cells into NOG mice. As shown in Fig. 1i/j, Lip-HNK treatment (20 mg/kg) drastically inhibited tumor growth. The combined treatment of Lip-HNK, CQ, and Carboplatin showed more superior antitumor effects (Supplementary Fig. 7a/b). In fact, we also compared Lip-HNK with standard chemistry in vitro, due to the limited number of mice, the limited nature of xenografts and the lack of intracranial tumorigenesis results, there was no statistical significance, but the combination group did have a better effect, at the same time, we also found that combination treatment for each drug significantly suppressed cell growth more than either single agent in vivo and in vitro (Fig. 1i–j and Supplementary Fig. 7a–c). At present, we have demonstrated that Lip-HNK alone or combined with chemotherapy (Carboplatin or Etoposide) causes significant regression of orthotopic xenografts (Fig. 1k and Supplementary Fig. 7d). The tumor tissue was associated with the elevated expression of cleaved caspase-3 and TUNEL-positive cells in the combination therapy group (Supplementary Fig. 7e–g). Taken together, CQ acting as an autophagy inhibitor combined with Lip-HNK may further improve the antitumor effect of Lip-HNK in vivo, Lip-HNK combined with chemotherapeutic drugs also improved the anti tumor effect.

All in all, Lip-HNK induced apoptosis and the ROS production, and inhibited the autophagy flux. Meanwhile, NAC, as a scavenger of ROS, can significantly block the generation and apoptosis of ROS induced by Lip-HNK (Supplementary Fig. 8). We also found that Lip-HNK did not damage the liver and kidney (Supplementary Fig. 9). These findings not only highlight the potential clinical application of Lip-HNK in the treatment of medulloblastomas, but also provide insights into the fundamental mechanisms of the anticancer effects of Lip-HNK.

## Supplementary information

3941R1_Supp.docx

Sigtrans_Supplementary_Materials_-marked-up version
