# Supplementary material for: Liposomal Honokiol induces ROS-mediated apoptosis via regulation of ERK/p38-MAPK signaling and autophagic inhibition in human medulloblastoma
Source: Signal Transduct Target Ther. 2022 Feb 21;7:49. doi: 10.1038/s41392-021-00869-w (PMC8858958; doi:10.1038/s41392-021-00869-w)
Supplement: Supplementary file 1 — 3941R1_Supp.docx [file 41392_2021_869_MOESM1_ESM.docx]

Supplementary Materials for

# Liposomal Honokiol Induces ROS-Mediated Apoptosis via regulation of ERK/p38-MAPK Signaling and Autophagic inhibition in Human Medulloblastoma

Shenglan Li^1#^, Jinyi Chen^1#^, Yaqiong Fan^1^, Ce Wang^1^, Can Wang^1^, Xiaohong Zheng^1^, Feng Chen^1*^and Wenbin Li^1*^

Correspondence to: chenfeng@bjtth.org and [liwenbin@ccmu.edu.cn](mailto:liwenbin@ccmu.edu.cn).

**This PDF file includes:**

Materials and Methods

Figures. S1 to S9

Materials and Methods

**Chemicals and Reagents**

Chengdu Jinrui Biotechnology Company (Chengdu, China) provided Lip-HNK (> 98% purity). We acquired N-acetyl-L-cysteine (NAC), DCFH-DA, and CCCP from Sigma-Aldrich (St. Louis, MO, USA). We purchased DMEM, fetal bovine serum, and 0.05% Trypsin-EDTA from Gibco BRL (Grand Island, NY, USA).

**Cell culture**

Medulloblastoma cells (DAOY and D283) and normal human hippocampal cells (HT22) were obtained from the American Type Culture Collection (ATCC). DAOY, BV2 and HT22 cell lines were cultured in DMEM, while D283 cell line was cultures in MEM medium (with 10% (vol/vol) FBS, which contained penicillin and streptomycin). Cells were placed in an incubator containing 5% carbon dioxide at a temperature of 37°C. The cells were grown to confluence before drug treatment.

**Western blot analysis**

After 48 hours of treatment, cells were harvest. Lysis of cells was conducted in RIPA buffer containing a mixture of phosphatase and protease inhibitors (Thermo Fisher Scientific, Waltham, Massachusetts, USA). The BCA™ Protein Assay Kit (Fremont Thermal Science, California, USA) was utilized to quantify the concentration of protein. The proteins were isolated using sodium dodecyl-polyacrylamide gel electrophoresis (SDS-PAGE), and bands were moved to a polyethylene fluoride (PVDF) membrane, which was then incubated overnight with the primary antibody blocking with 5% milk at 37°C for 60 mins, then with the secondary antibody. Finally, the ECL system (Perkin Elmer, Waltham, MA, USA) was used to detect immune complexes. Antibodies against the following proteins were used: Bcl-2(1:1000), Bax(1:1000), cyclin D1(1:1000), cleaved caspase-3(1:1000), caspase-3(1:1000), ERK(1:1000), p-ERK(1:1000), p38(1:1000), p-p38(1:1000) , CDK4(1:1000), and GAPDH(1:1000) (Cat. #15071, #5023, #55506, #9661, #9662, #4695, #4370, #8690, #14451, #12790, #5174, Cell Signaling Technology, Danvers, MA, USA). The secondary antibodies (1:5000) were from Abcam (Cambridge, UK).

**Cell viability assay**

CCK-8 assay was applied to determine cellular vitality. The microplate reader Synergy™ was used to measure the 450 nm absorbance after incubation for 60 min.

**Colony formation assay**

For colony-forming analysis, 1.0x10^3^ DAOY cells were seeded into 6-well plates and incubated with different concentrations of Lip-HNK. After culturing for 14 days, the cells were fixed with 4% formaldehyde and then stained with 0.5% crystal violet for 15min to visualize and quantify the clones.

**Cell cycle analysis**

DAOY and D283 cells were seeded into a 6-well plate at a density of 5×10^5^ cells/well and treated for 48 h with Lip-HNK at various concentrations. the PI fluorescence could be measured and analyzed using a flow cytometer.

**Staining with Hoechst 33342**

Cells were pretreated with different concentrations of Lip-HNK. After fixed with cold methanol, they were stained with Hoechst 33342 and the morphological characteristics of apoptotic cells could be observed by fluorescence microscopy.

**Apoptosis assay with annexin V and PI staining**

An apoptosis test kit (Annexin V-PI: BD Biosciences, San Jose, CA, USA) was used to investigate apoptosis.

**Mitochondrial membrane potential assay**

MMP was measured using the JC-10 assay kit (Solarbio, Beijing, China). MMP changes were monitored based on the fluorescence intensity using flow cytometry. The positive control was treated with CCCP.

**Hoechst 33342 and PI Staining**

Cell mortality was examined using Hoechst 33342 (1 μg/mL) and PI (1 mg/mL) staining. The mortality rate (%) was determined as the proportion of PI-positive and Hoechst staining-positive cells.

**Measurement of intracellular ROS generation**

As described previously^1,2^, DCFH-DA (Sigma, MO, USA) could be used to measure ROS production. a flow cytometer (BD Biosciences, San Jose, California, USA) was used to measure DCF fluorescence, and FlowJo10 was used to analyze the data.

**Acridine orange staining**

In this study, acridine orange staining was used to analyze the formation of acidic vesicular organelles (AVOs)^3^. DAOY cells were observed under fluorescence microscope and analyzed by flow cytometry.

**Transmission electron microscopy observation**

Changes in cell ultra-structure caused by HNK were visualized using transmission electron microscopy (TEM). Autophagy was evaluated by examining autophagosome formation. The treated cells were fixed with 2.5% glutaraldehyde and post-fixed with 1% osmium tetroxide. After being dehydrated in increasing concentrations of alcohol, the cell pellets were embedded in epon. Representative areas were chosen for ultrathin sectioning and examined on a transmission electron microscope at a magnification of ×5000.

**Tumor Xenograft Studies**

The NOG mice were reared in a specific pathogen-free environment. 200 μL（PBS: Matrigel=1:1) DAOY cells (5×10^6^) were subcutaneously injected into the right side of each mouse. Seven days later, the mice were randomly divided into vehicle group, Lip-HNK group, CQ group and Lip-HNK + CQ group (4 mice in each group). The vehicle group was given empty liposome, and the treatment group was injected with Lip-HNK (20 mg / kg), CQ (50 mg/kg) or the combination of the two drugs. Lip-HNK was injected intraperitoneally 6 times a week and CQ was injected intraperitoneally 3 times a week. The body weight and tumor volume were measured six times a week. The mice were sacrificed 35 days later. All experimental procedures were approved by animal ethics committee of Beijing Tiantan Hospital.

**PDX Medulloblastoma Mouse Models**

Studies were conducted in PDX medulloblastoma mouse models, pediatric patient tumor tissue obtained from Beijing Tiantan Hospital with approval from the Institutional Review Board. It is a MYC-amplified Group 3 medulloblastoma with large cell/anaplastic morphology from a 3-year-old patient. Tissue was first xenografted into mice within hours of surgical removal from the patient, and passaged exclusively in immunocompromised mice (NOG).

**Orthotopic Xenografts**

Tissue was harvested from intracranial tumors in symptomatic donor mice and processed in serum-free Dulbecco’s Modified Eagle’s Medium (DMEM) or phosphate buffered saline (PBS) by trituration through an 18-g needle. The cells were filtered, centrifuged, and resuspended in serum-free DMEM or PBS. For orthotopic xenografts athymic mice were anesthetized and an incision was made to expose the skull. A hole was created in the calvarium above the right cerebellar hemisphere, two mm lateral (right) to the sagittal suture, two mm posterior of the lambdoid suture, using a microdrill (0.9 mm burr). 5 ul of cell suspension (100,000 cells) was injected into the brain parenchyma approximately 2 mm under the dura. The burr hole site was filled with SurgiFoam and the incision was closed with tissue glue.

**MRI**

Mice were enrolled when they developed signs of brain tumor development (mild to moderate head bulge) and assigned to different treatment. MRI was performed using Tesla (T) Bruker ICON mouse imaging system (Aspect Imaging, Shoham, Israel).

**Histology TUNEL staining**

TUNEL staining was performed according to the manufactured instructions.

**Statistical analysis**

One-way ANOVA was used to analyze the experimental data using SPSS version 19.0 software (SPSS Inc., Illinois, USA), the results obtained were expressed as mean ± standard deviation (SD). A p-value < 0.05 was considered statistically significant.

**Reference**

1 Eruslanov, E. & Kusmartsev, S. Identification of ROS using oxidized DCFDA and flow-cytometry. *Methods Mol Biol*. **594**, 57-72, (2010).

2 Wang, X. & Roper, M. G. Measurement of DCF fluorescence as a measure of reactive oxygen species in murine islets of Langerhans. *Anal Methods*. **6**, 3019-3024, (2014).

3 Degtyarev, M., Reichelt, M. & Lin, K. Novel quantitative autophagy analysis by organelle flow cytometry after cell sonication. *PLoS One*. **9**, e87707, (2014).


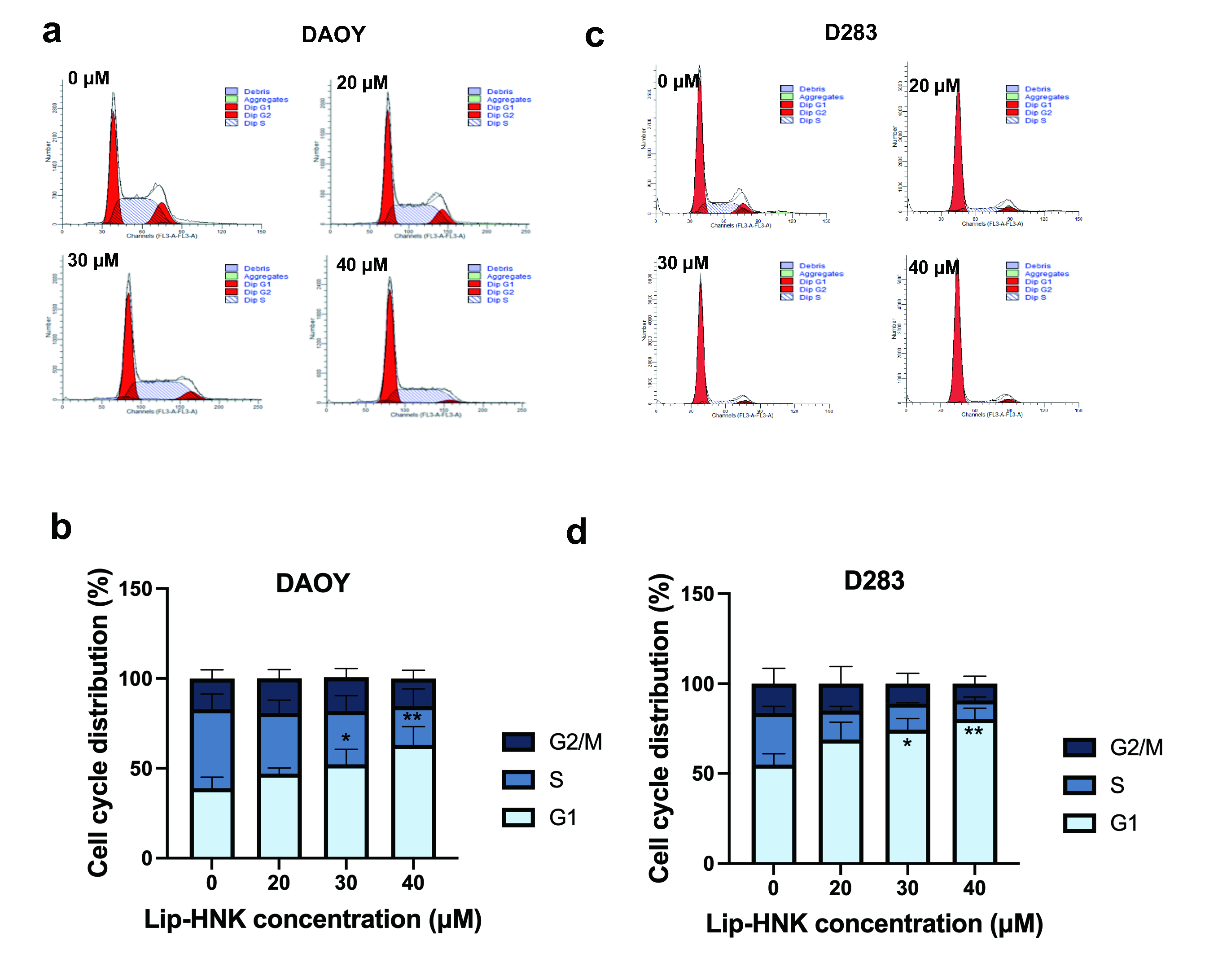


**Supplementary Figure 1. Lip-HNK-induced cell cycle arrest in medulloblastoma cells.** (a, c) Processed cell cycles of DAOY and D283 cells after 48 h for treatment with 0, 20, 30, and 40 μM Lip-HNK. (b, d) Flow cytology, of PI-stained cells for quantitative analysis at each cell cycle stage. The experiment was replicated three times. We collected data from at least three independent experiments, and values are presented as the mean ± SD. *: p < 0.05, **: p < 0.01.


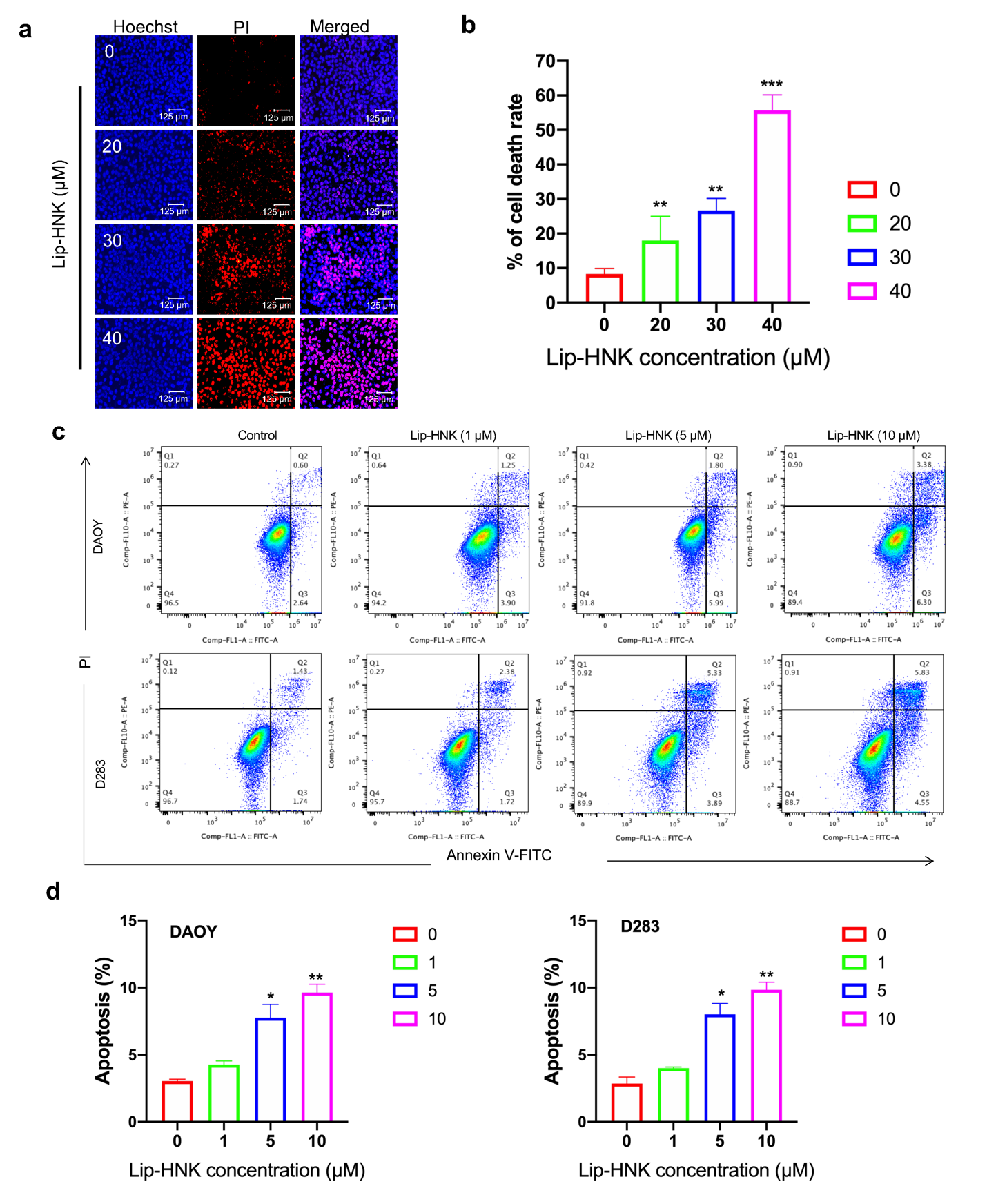


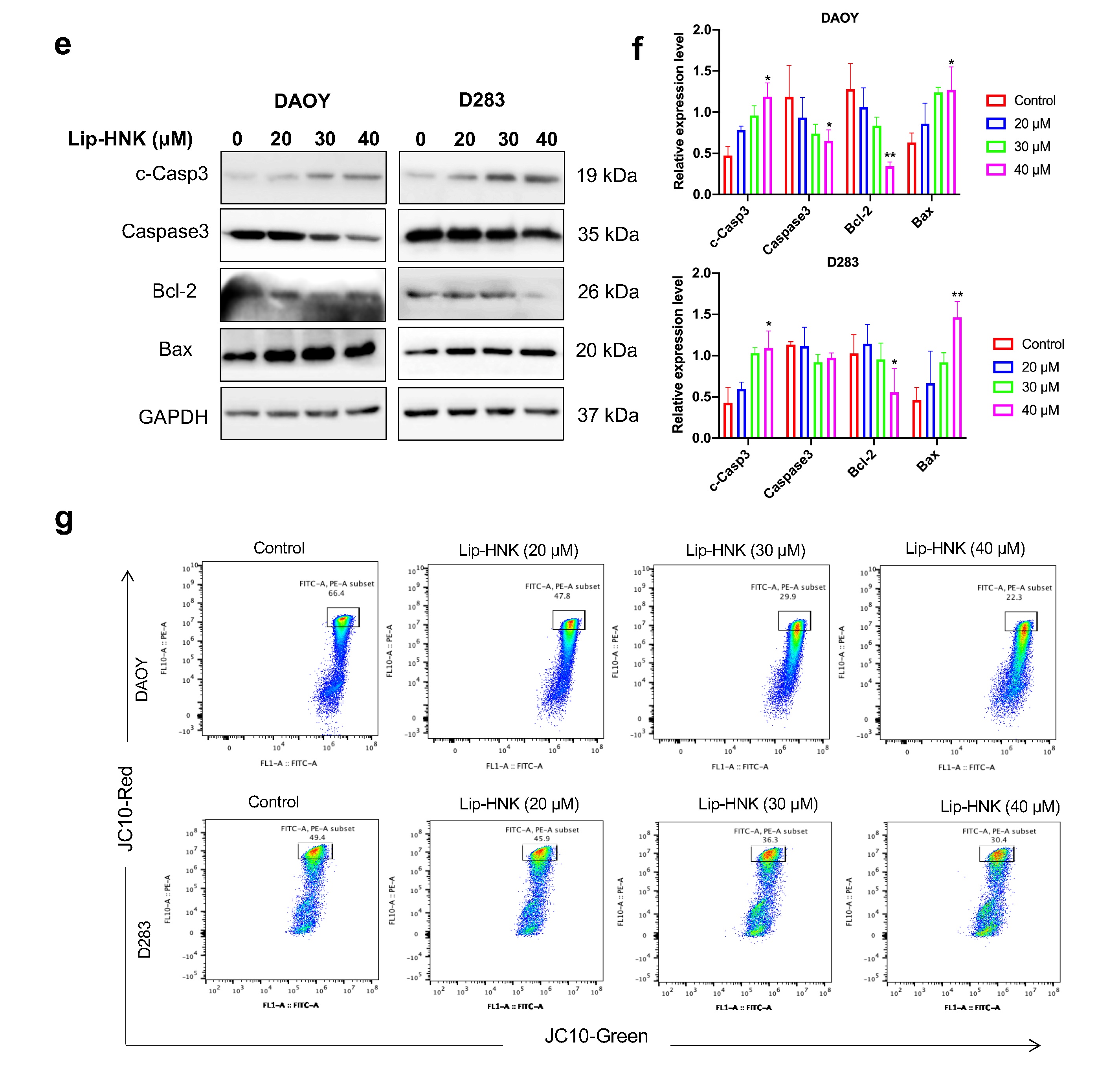


**Supplementary Figure 2. Lip-HNK promoted apoptosis of medulloblastoma cells.** (a) The nuclear structure of Hoechst 33342/PI-stained DAOY cells was analyzed using a fluorescence microscope. (b) The ratio of PI stained (red) to Hoechst 33342 stained (blue) cells reflected cell apoptosis. (c) Determination of apoptotic cells via annexin V-FITC/PI flow cytology analysis. Results represent three independent experiments. (d) The percentage of apoptotic cells was shown. The data are presented as the mean of three independent experiments. (e) Western blots showing expression of apoptosis proteins Bcl-2, Bax, Caspase-3, and cracked Caspase-3. (f) Data was obtained from at least three separate experiments. (g) Flow cytology, with red/green fluorescence analysis, for MMP stained with fluorescent mitochondrial probe JC-10.


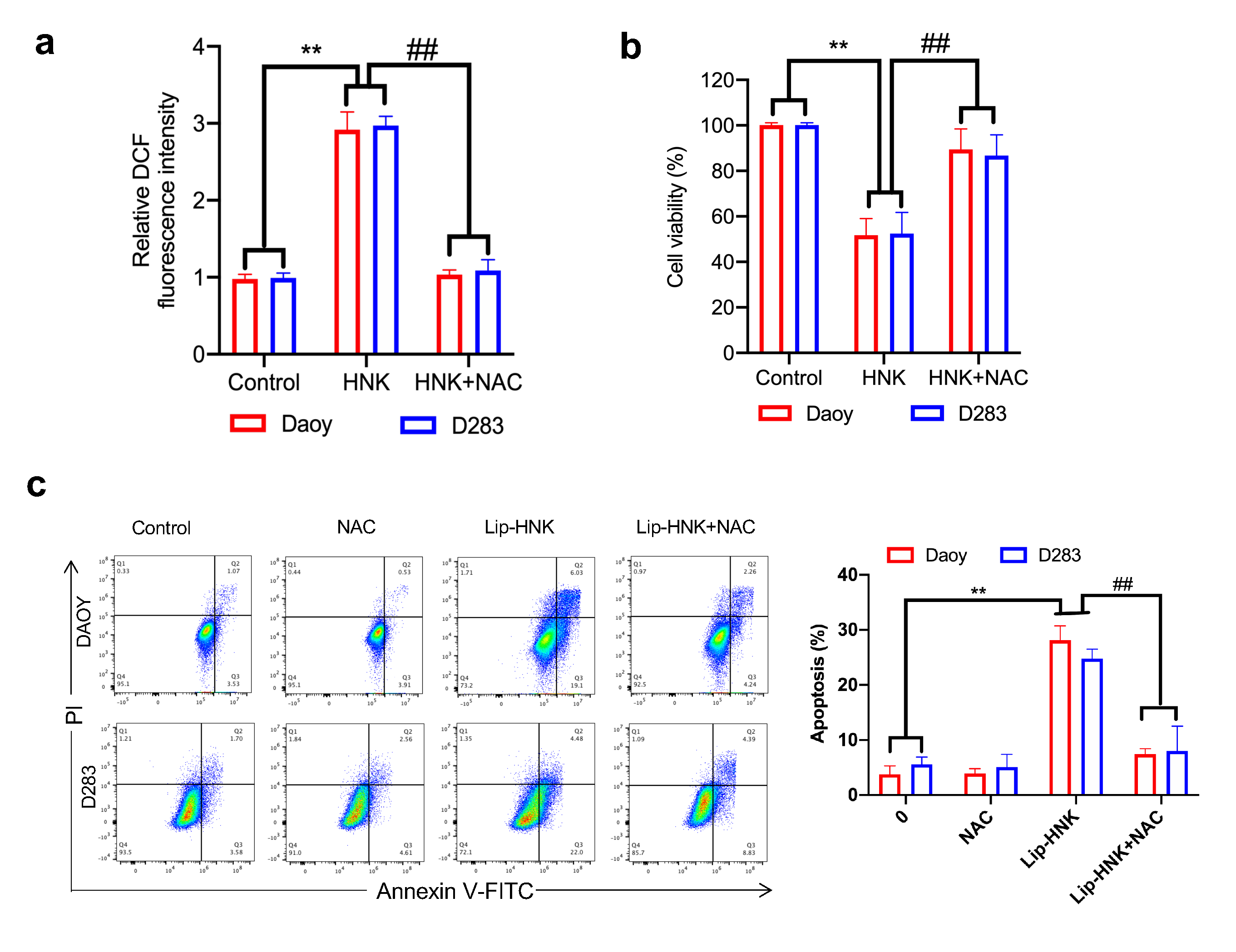


**Supplementary Figure 3. ROS in Lip-HNK human medulloblastoma cells affect Lip-HNK-induced apoptosis.** (a) Relative DCF fluorescence intensity presented as the multiple of DCF fluorescence relative to the control. The percentage of cell survival rate determined using CCK-8 is also illustrated in the histogram (b). (c) Using flow cytometry, cells can be stained with annexin V-FITC/PI assay. We collected data from at least three independent experiments, and values are presented as the mean ± SD. *: p < 0.05, **: p < 0.01 versus the control group.


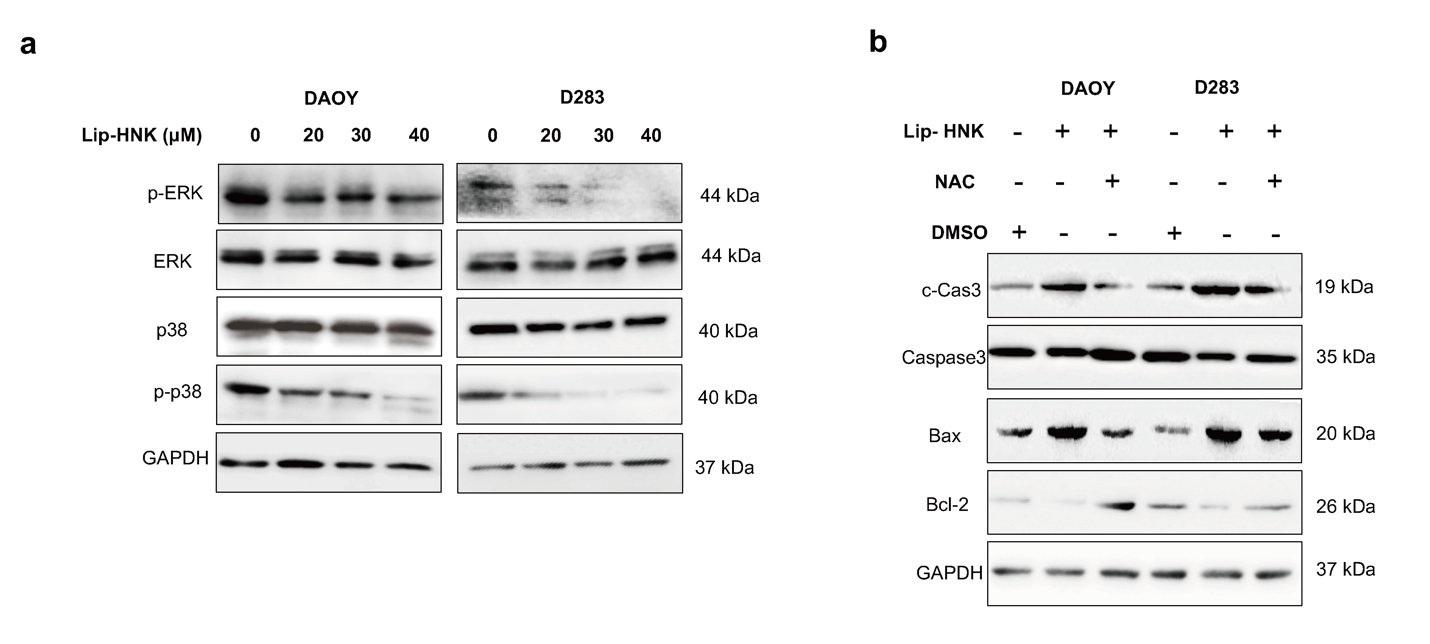


**Supplementary Figure 4. Inhibition of the ERK/p38 MAPK signaling pathway in medulloblastoma cells under Lip-HNK treatment.** (a) Levels of p-ERK, ERK, p-p38, and p38 proteins for medulloblastoma cells treated with 0, 20, 30, and 40 μM Lip-HNK for 48hrs. (b) Levels of apoptosis-related factors, cleaved caspase 3, caspase 3, Bax, and Bcl-2, measured via immunoblotting.


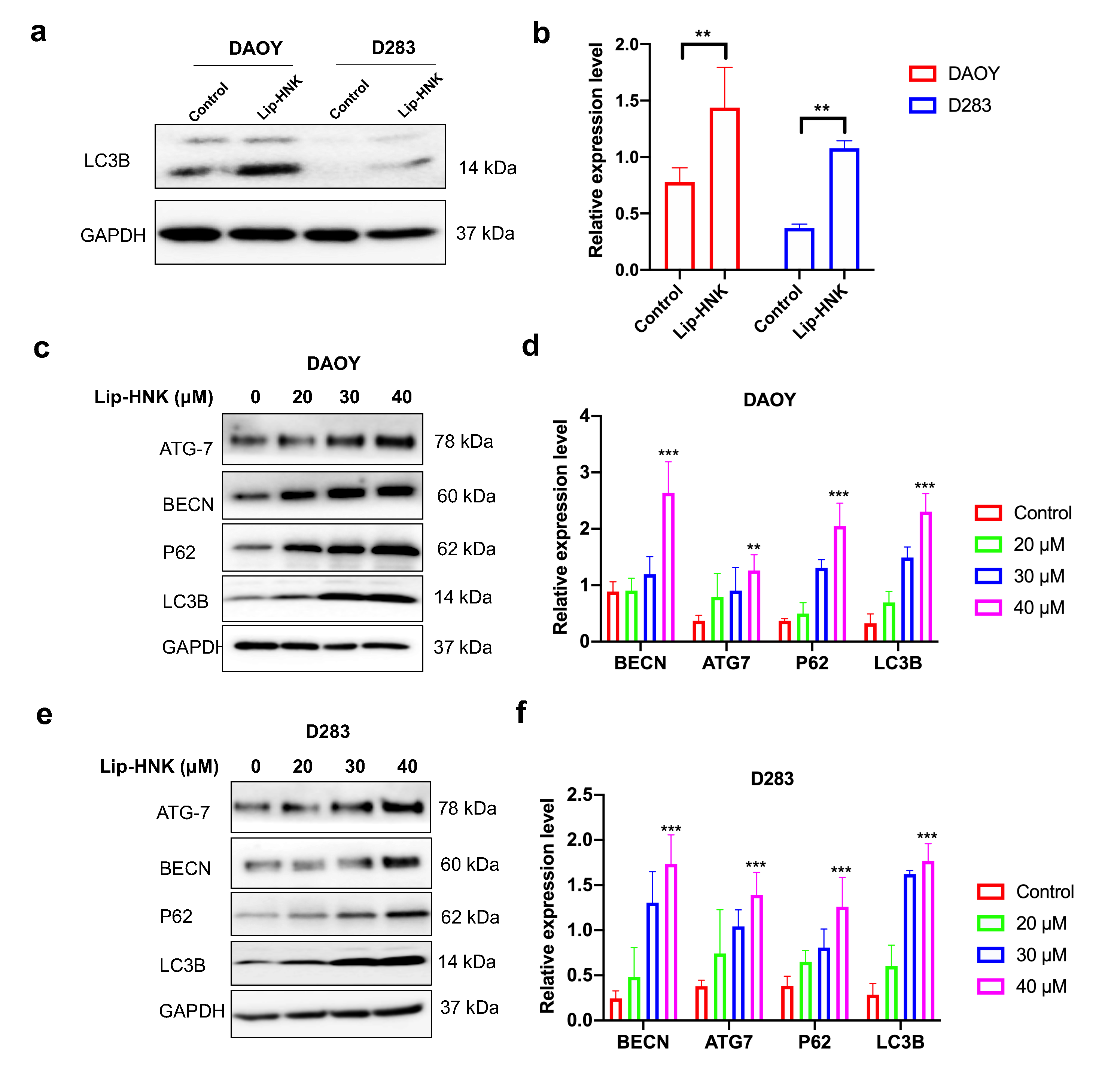


**Supplementary Figure 5. Lip-HNK treatment encourages early autophagy, but inhibits autophagy flux.** (a) Western blot was used to investigate the expression level of LC3B in DAOY and D283 cells. (b) The relative expression levels of LC3BII protein. Western blot was performed in DAOY (c) and D283 cells (e) with antibodies against LC3B, BECN, Atg7, P62, and GAPDH. The relative protein expression level is illustrated in the histogram (d, f). All bar graph statistics are shown as mean ± SD. *: P < 0.05. **: P < 0.01. ***: P < 0.001.


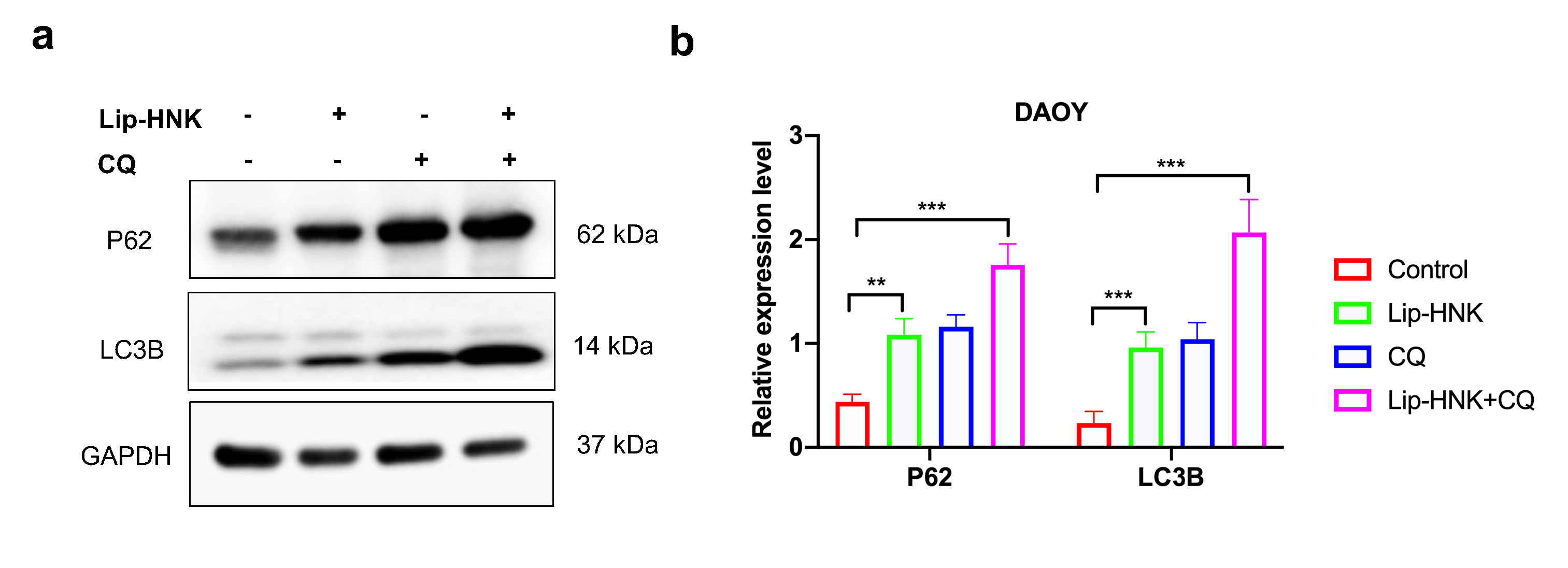


**Supplementary Figure 6．Lip-HNK inhibits the fusion of autophagosome and autophagosome.** (a) DAOY cells were treated with Lip-HNK with or without CQ (10 μM). Western blotting analysis was used to investigate the protein levels of LC3B and p62. (b) Results were normalized to GAPDH. The data were expressed as mean ± standard deviation (SD). **Compared with Lip-HNK treated cells, P < 0.01；***Compared with Lip-HNK treated cells, P < 0.001.


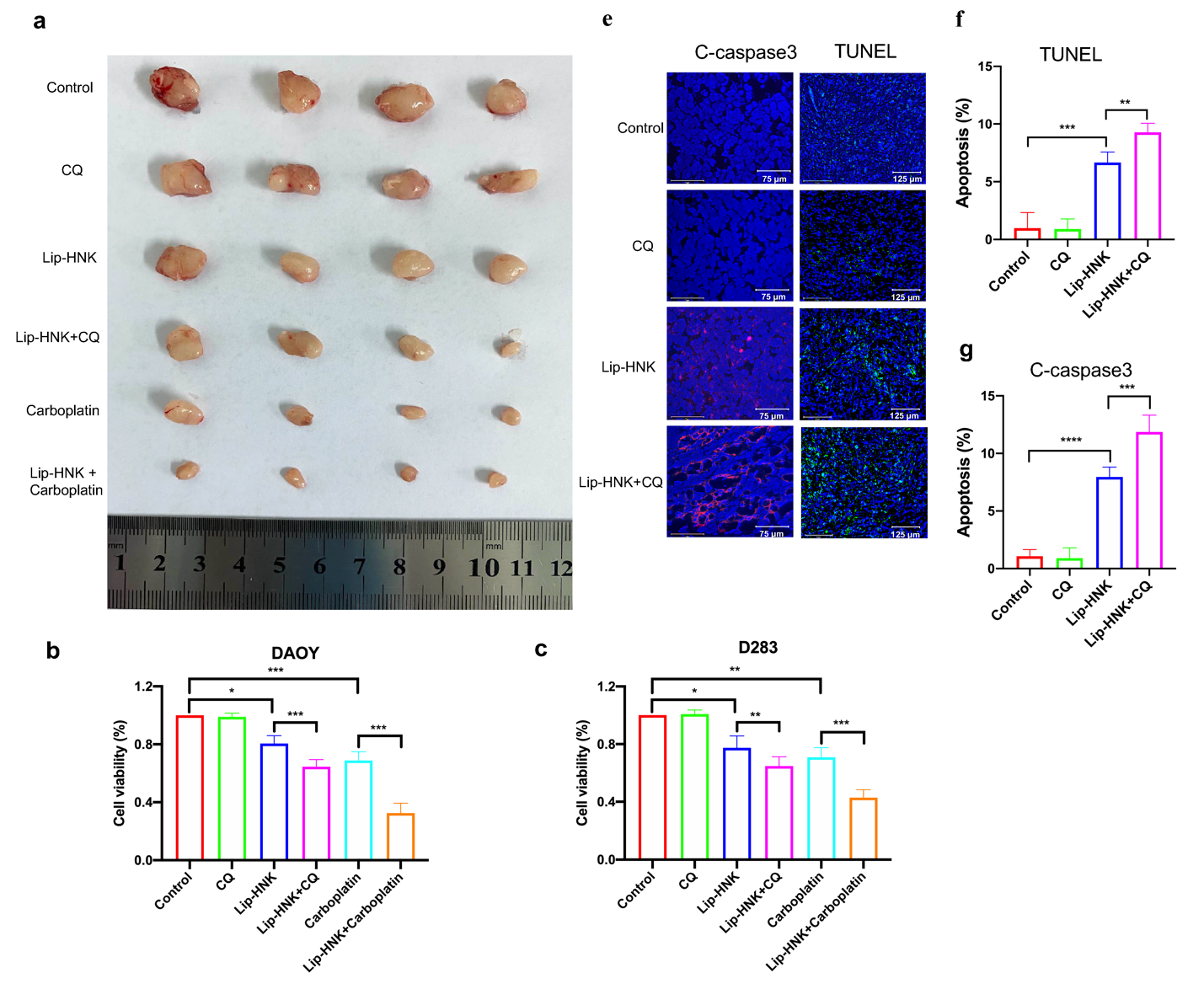


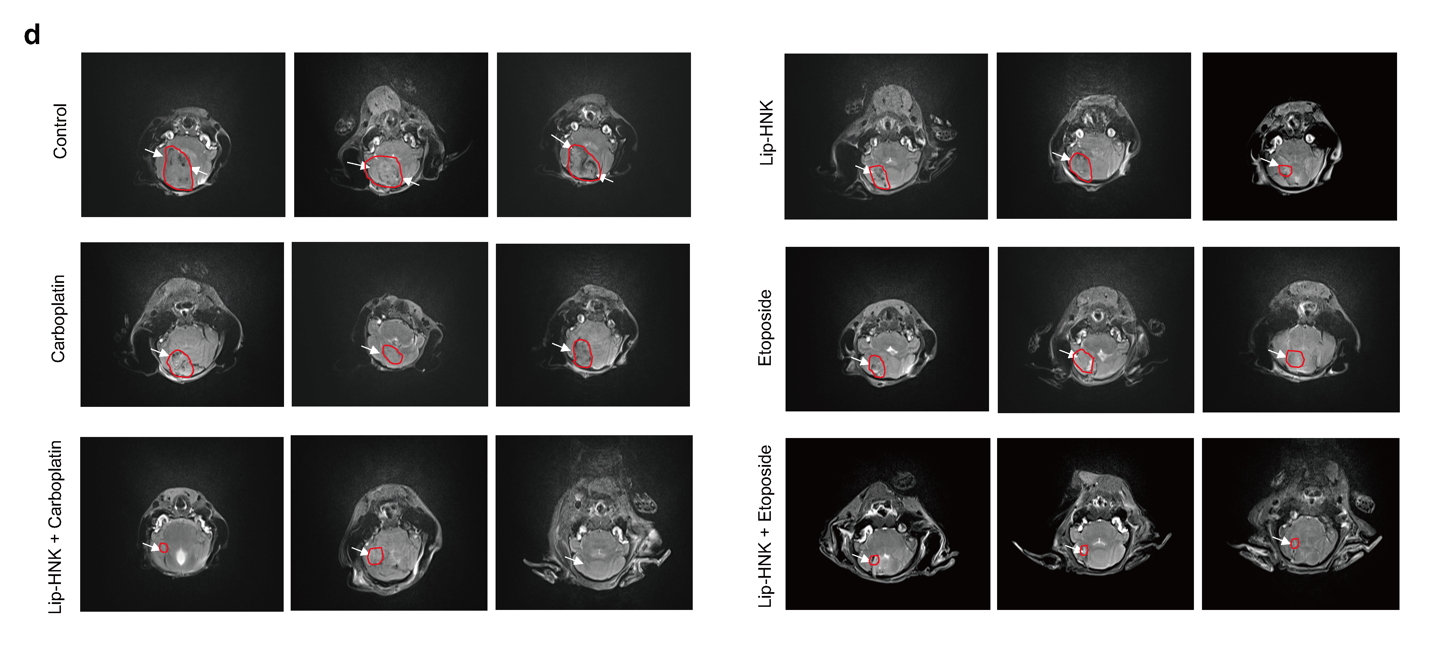


**Supplementary Figure 7. CQ can improve the antitumor effect of Lip-HNK in medulloblastoma xenograft models and coronal MRI study demonstrates that Lip-HNK alone or combined with chemotherapy causes significant regression of brain tumors.** The xenograft tumors of NOG mice were treated with intraperitoneal injection of empty liposomes, CQ (50 mg/kg), Lip-HNK (20 mg/kg), and Lip-HNK combined with CQ. After 28 days of cell injection, the mice were sacrificed and tumor samples were collected. (a) Representative images of tumor size in different groups. Growth assay performed at the IC25 for Lip-HNK and carboplatin shows combinatorial efficacy in DAOY (b) and D283 (c) medulloblastoma cells. (d) The representative image of intracranial tumors in each group was shown. The dose of Carboplatin was 50 mg/kg and Etoposide was 10 mg/kg. After daily i.p. injected Lip-HNK (20 mg/kg) for 21 days, the anatomical images of the intracranial tumors were visualized by small animal MRI scanner. (e) The apoptosis of tumor cells was detected by cleaved-caspase-3 immunofluorescence and TUNEL staining. (f) TUNEL positive and (g) cleaved caspase 3 positive cells have been quantified. Data is presented as mean ± SD. **: p < 0.01, ***: p < 0.001, ****: p < 0.0001 versus control.


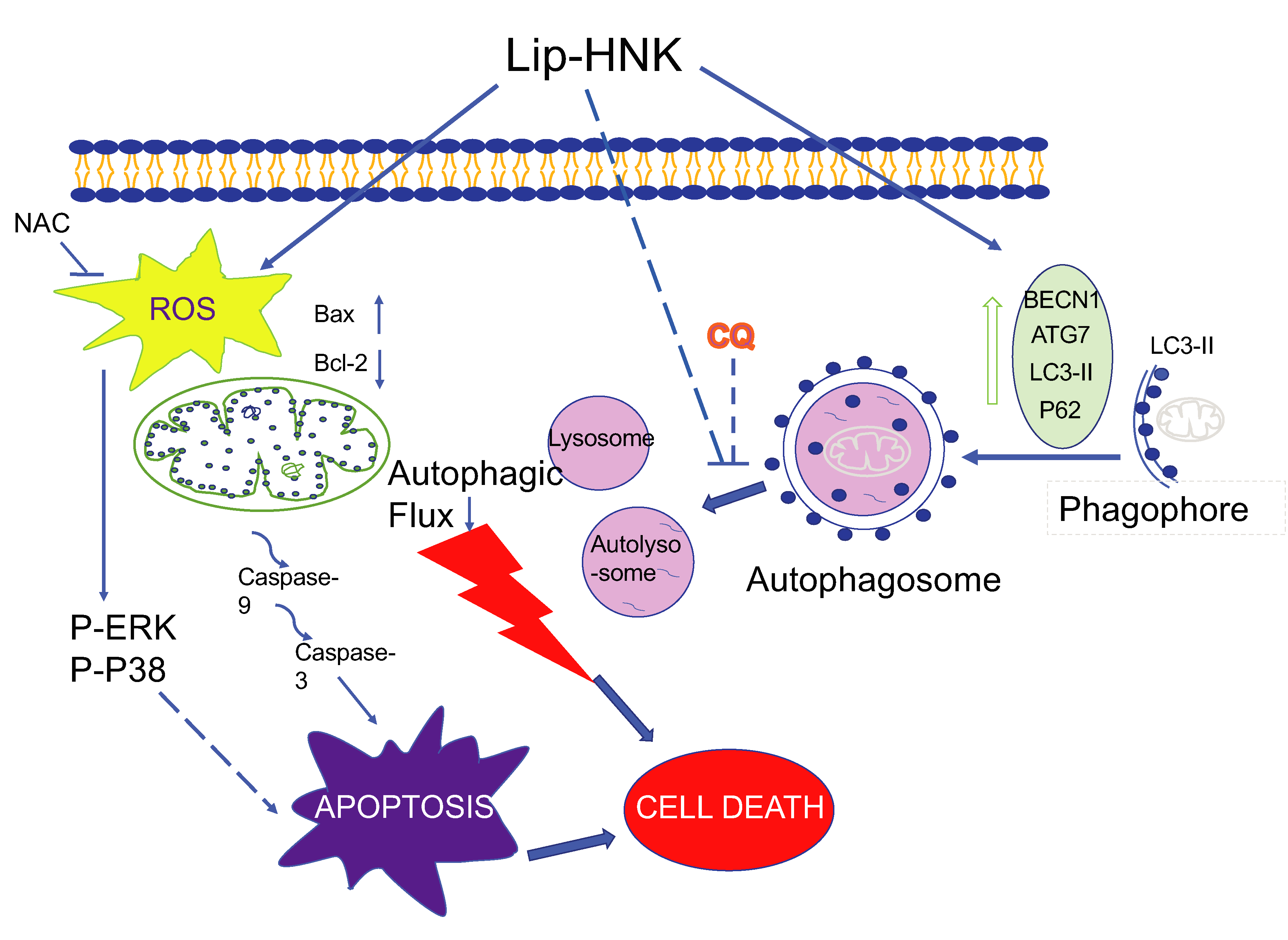


**Supplementary Figure 8．The mechanism of Lip-HNK-induced apoptosis in medulloblastoma cells is illustrated.**


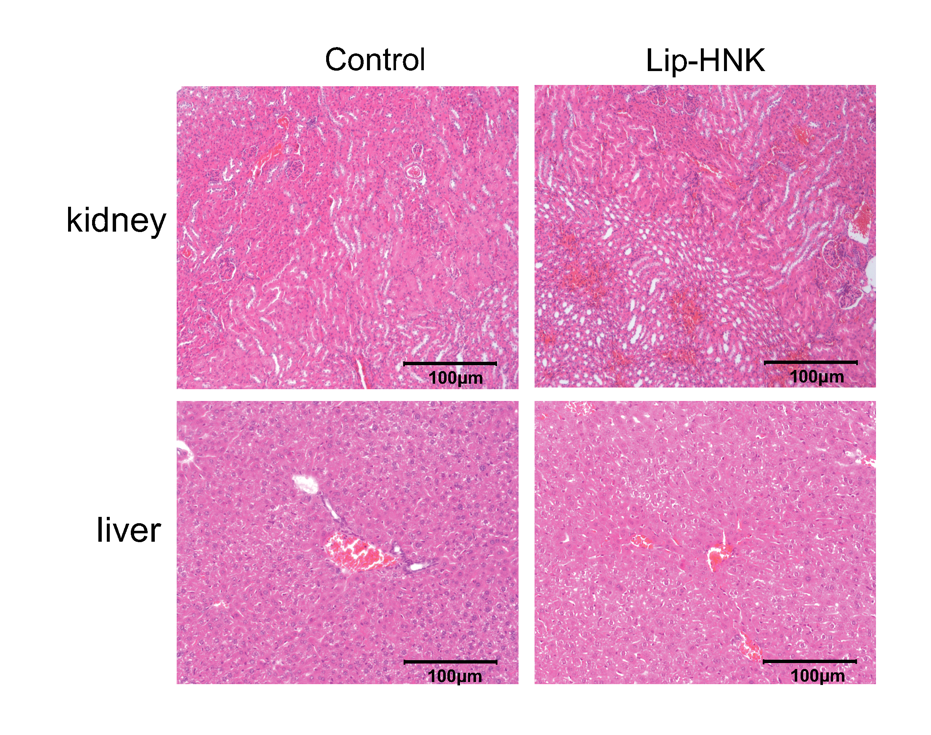


**Supplementary Figure 9．HE staining showed that Lip-HNK did not damage liver and kidney tissue.** Scale bars, 100 μM.
